# Response to Climate Change and GAP Analysis of *Thuja koraiensis* Nakai

**DOI:** 10.3390/plants13131750

**Published:** 2024-06-25

**Authors:** Xiuhua Yang, Xiaoyu Li, Jiaqi Cui, Ruiqi Liu, Jitong Li, Chengjun Yang

**Affiliations:** Northeast Asia Biodiversity Research Center, College of Forestry, Northeast Forestry University, Harbin 150040, China; 2022120179@nefu.edu.cn (X.Y.); xiaoyu_li2022@163.com (X.L.); 2022120205@nefu.edu.cn (J.C.); 2021125110@nefu.edu.cn (R.L.); lijitong101@163.com (J.L.)

**Keywords:** *Thuja koraiensis* Nakai, Maxent model, Marxan model, potentially suitable area, GAP analysis

## Abstract

Due to global warming and increased human activity, the wild population of *Thuja koraiensis* Nakai (*T. koraiensis*) has dropped, placing it in danger. An understanding of the response of *T. koraiensis* to climate change and the determination of priority conservation areas are tremendously critical for proper conservation. Using sixty-nine *T. koraiensis* distribution points and seven environmental factors, the Maxent model was used to predict potentially suitable areas and spatial variation patterns of *T. koraiensis* and the Marxan conservation planning model was used to evaluate conservation gap areas. Research shows that the dominant environmental factors affecting the distribution of potentially suitable areas for *T. koraiensis* included elevation, precipitation of the driest month, isothermality and precipitation of the wettest quarter. Under the current climatic conditions, highly suitable areas for *T. koraiensis* are mainly distributed in the Changbai Mountains within Samjiyon County and Baishan City, the Hamgyong Mountains within the western part of Hamgyong-Bukto Province, and the T’aeback-Sanmaek Mountains within Gangwon-do, Kumgangsan Special Administrative Region and Kangwon-do. Under future climate conditions, suitable areas for *T. koraiensis* show a decreasing trend, and the suitable area will be reduced to higher elevations, and the Hamgyong Mountains may become a refuge. Based on GAP analysis, 69.69% of the priority conservation areas of *T. koraiensis* are located outside of the nature reserve, and these conservation gap areas are primarily in the southern part of the Changbai Mountains and Kangwon-do.

## 1. Introduction

Global climate change has already threatened biodiversity [1]. According to the sixth assessment report of the Intergovernmental Panel on Climate Change (IPCC), the average temperature of the Earth’s surface from 2011 to 2020 was 1.1 °C higher than the average temperature at the end of the 19th century (before the Industrial Revolution). Due to human activities, the content of greenhouse gases in the atmosphere continues to increase, thus exacerbating the trend of global warming [2]. Global climate changes have destroyed the habitats of numerous species, thus resulting in ecosystem degradation and the risk of extinction of numerous species [3]. In particular, endangered plants are more sensitive to global climate change [4]. Hence, biodiversity conservation is extremely important for studying the distribution of endangered plants in suitable areas under different climate scenarios to provide a theoretical and practical basis for formulating effective species protection measures [5].

Species distribution models are based on distribution data and related environmental data, and the ecological requirements of species are calculated according to certain algorithms to predict the potentially suitable area distributions of species [6]. The species distribution model is applied for determining the effects of climate change on species [7], assessing the risk of invasive species [8], controlling pests [9], and protecting animals and plants [10]. At present, algorithms for predicting potentially suitable areas for species include the Maxent (max entropy), GARP (genetic algorithm for rule-set production), BIOCLIM (bioclimatic modeling), and Domain (domain-environmental envelope) algorithms [11]. Although the spatiotemporal extrapolation ability of the Maxent model is better only under low-threshold conditions (when there is data deficiency or scarce samples), the model can still maintain relatively high accuracy; thus, the Maxent model is usually regarded as the first-choice model for the prediction of endangered plants [12,13,14].

Systematic conservation planning is a regional biodiversity conservation planning method that was developed using analysis of biodiversity conservation and GAP analysis based on ecological zones. This method can not only achieve the goal of biodiversity conservation but can also solve the contradiction of the human–land relationship (that is, under certain temporal and spatial conditions, the opposition and unity between the requirements of human survival and development and the geographical environment in terms of space and the exchange relations of matter, energy and information) and promote the sustainable development of biodiversity [14,15,16]. The Marxan model is a relatively widely applied systematic conservation tool. Although this tool has limitations (specifically, it cannot easily integrate random or time-dependent data), it can identify priority conservation areas on the basis of fully considering the socioeconomic factors of the conservation system and can effectively improve conservation benefits [17,18].

GAP analysis is a method that uses geographic information systems to perform spatial analysis of the distribution and conservation status of various elements of biodiversity to determine the conservation gaps of vegetation types and endangered species that do not appear in the biodiversity conservation area network. Moreover, this analysis can be used to implement compensatory measures by changing land management or building new protected areas [19]. This method can analyze and evaluate the current situation of biodiversity conservation and the protection effect of existing protected areas [20]. It has been applied to GAP research and the analysis of endangered species [21], narrowly distributed species [22], mammals [23], birds [24], and ecosystems [25], among other factors.

*T. koraiensis (Thuja koraiensis* Nakai) is an evergreen tree that is distributed in the Changbai Mountains of China and the Korean Peninsula. It is a vulnerable indicator plant of global warming and a protected species with an extremely small population [26,27]. It is a valuable plant resource that integrates ecological, landscaping, timber and economic values [28]. However, due to climate change and excessive human deforestation, the living environment of *T. koraiensis* has been destroyed, and the number of wild *T. koraiensis* has dramatically decreased, thus leading to its near extinction [29]. Studies on *T. koraiensis* have mainly focused on tree height growth [30], population surveys [31], genetic research [32], breeding methods [33], wood structure [34], essential oil research [35], and suitable area prediction [36,37], among other factors. However, the combination of the Maxen model and Marxan model for planning conservation areas for *T. koraiensis* has not been reported.

Therefore, this study used the Maxent model to predict the current and future potentially suitable areas for *T. koraiensis*. Furthermore, the planning unit versus the conservation feature file is made based on the distribution probability of the potentially suitable areas under the current climatic conditions, and the Marxan model is used to identify its priority conservation areas. The purpose of this study was to (1) predict the potentially suitable areas of *T. koraiensis* under current and future climatic conditions and analyze its spatial pattern changes; (2) explore the dominant environmental factors affecting the distribution of *T. koraiensis*; and (3) identify conservation priority areas and evaluate the effectiveness of conservation of *T. koraiensis*. The research results can provide reasonable guidance for the planning of *T. koraiensis* reserves.

## 2. Materials and Methods

### 2.1. Data Sources and Screening

**Figure 1:**
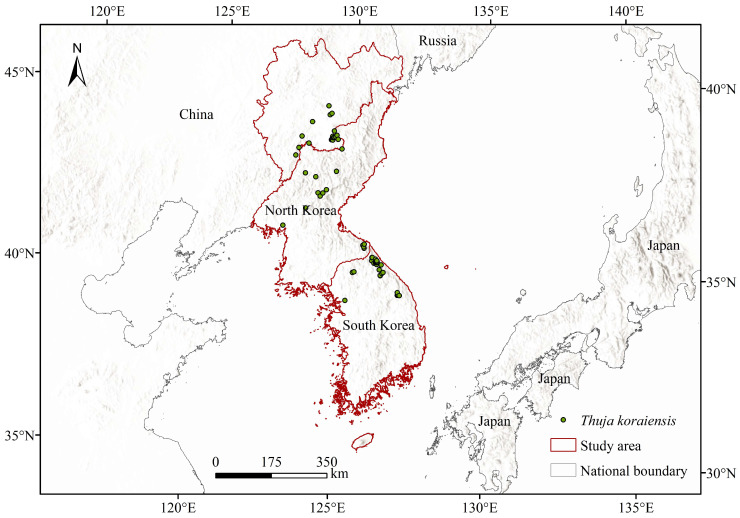
Distribution points of *T. koraiensis*.

All of the data sources that were used in this study are listed in Table 1. The distribution data of *T. koraiensis* were obtained from the Chinese Virtual Herbarium database, the Global Biodiversity Information Facility and related literature [36]. The ENMTools tool was used to remove repeated coordinate points in the same grid, and 69 distribution points of *T. koraiensis* were ultimately obtained (Figure 1).

The environmental data that were used in the Maxent model included 19 climatic factors and 1 topographic factor. The climate data of the 2050s (2041–2060) and 2090s (2081–2100) in the future time period were selected from the four climate scenario datasets of SSP126, SSP245 and SSP585 under the BCC-CSM2-MR model in the sixth international coupled model comparison plan (CMIP6). To avoid model overfitting caused by multicollinearity among environmental factors, this study used ENMTools 1.3 software to perform a Pearson correlation analysis. When the absolute value of the correlation coefficient is greater than 0.85, the environmental factors with a small relative contribution rate to the model prediction are eliminated [38]. Finally, data from 6 climatic factors and 1 topographic factor were selected for modeling.

### 2.2. Maxent Model Construction

The Maxent model estimates a set of functions that correlate environmental variables with distribution probabilities based on the maximum entropy principle of existing data to obtain the niche and potential geographical distribution of approximate species. In this study, Maxent 3.4.4 software was used to predict the potentially suitable areas for *T. koreanum* in three time periods. Based on the distribution point data and environmental variable data, the ENMeval package was used to optimize the regularization multiplier (RM) and feature combination (FC). The RM parameter value was 0.5–4, and one value was taken at every interval of 0.5, for a total of 8 parameter values. The features of the Maxent model include linear (L), quadratic (Q), product (P), threshold (T) and hinge (H) features. In this study, six feature combinations (‘L’, ‘H’, ‘LQ’, ‘LQH’, ‘LQHP’ and ‘LQHPT’) were selected for testing. Finally, the Akaike information criterion correction (AICc) was used to test the fitting degree and complexity of the Maxent model. An AICC of 0 was considered to be the best parameter combination [39]. Using Maxent software, 75% of the geographical distribution points were selected as training data, and 25% were selected as test data [40]. The type of repeated operation was set to bootstrap, and 10 replicates were used for modeling. The area under the curve (AUC) under the receiver operating characteristic curve (ROC) was used to evaluate the simulation accuracy of the Maxent model. A larger AUC value indicates a higher simulation reliability [41]. To evaluate the performance of the Maxent model, this study also used the ENMeval package to construct a null model, and the number of iterations of the null model simulation was set to 100 [42].

### 2.3. Marxan Model Construction

The Marxan model uses the simulated annealing algorithm to identify the lowest cost planning unit combination that can achieve the protection goal in the conservation system through iterative operation [43]. In this study, the Marxan model was used to identify the conservation priority areas of *T. koraiensis*. In the preparation of the input file, the study area was divided into 39,137 planning units with an area of 9 km^2^. The intensity of human activities is a comprehensive indicator that represents the degree of influence of human activities on the surface of the earth [44]. Therefore, the intensity of human activities is taken as the protection cost of every planning unit. When quantifying the intensity of human activities, the road density, the percentage of global impervious surface area, the population density, and the landscape development intensity index (LDI) were selected to determine the comprehensive human activity intensity (HAI) [17,45]. The calculation formula is displayed below:

HAI=∑i=1 nXi×Wi

In the formula, $X_{i}$ the denotes standardized value of index i and the $W_{i}$ weight of index i. The weight is calculated by the entropy weight method [46]. The calculation steps are as follows: (1) data standardization of indicators; (2) calculate the entropy value of the index; (3) calculate the entropy weight. The weights of these four indicators are road density (0.41), global impervious surface area percentage (0.28), population density (0.1) and landscape development intensity index (0.21).

The LDI is a potential human disturbance index based on land use and can be calculated based on the LDI coefficient in Table 2 [47] as follows:

$${LDI}_{total}=\sum \%LU_{i}\;\times \;{LDI}_{i}$$

where

${LDI}_{total}\;$= LDI ranking for landscape unit
${\%LU}_{i}$ = percent of the total area of influence in land use i
${LDI}_{i}$ = landscape development intensity coefficient for land use i.

With reference to the Aichi Biodiversity Target of protecting at least 17% of land and inland water as standard, this case study set the protection target for *T. koraiensis* at 20%. SPF (species penalty factor) is the penalty scale that the model will allocate to the objective function when the conservation target is not met [48]. Each simulation operation modified the boundary length modifier (BLM) parameters to identify the most suitable point for ensuring the tightness of the conservation system to avoid excessive density or excessive dispersion of the conservation system [49]. We combined the ZonaeCogito 1.74 software and the ArcMarxan toolbox to optimize the parameters of the Marxan model and obtained the optimal species penalty factor (SPF) and BLM values of 2.4 and 0.00000342, respectively. The model was iterated 100 times, and the area with high irreplaceability of the planning unit was obtained as the conservation priority area.

**Table 2:** Land use type and its corresponding LDI coefficients.

Land Use Type	LDI Coefficient
Cultivated land	4.54
Forest	1.58
Grass land	2.77
Shrubland	1.58
Wetland	1
Water body	1
Artificial surfaces	8.66
Bareland	6.92
Permanent snow and Ice	1

### 2.4. Data Analysis and Processing

The potentially suitable areas output data via the Maxent model were imported into ArcGIS for visual analysis. The natural discontinuity classification approach was utilized to divide the suitable area of *T. koraiensis* into four grades: no suitability (0–0.07), low suitability (0.07–0.21), medium suitability (0.21–0.47) and high suitability (0.47–1.00) [50]. Moreover, the SDM toolbox was utilized to calculate the spatial variation pattern of suitable areas of *T. koraiensis* under diverse climate scenarios. The priority conservation areas identified by Marxan were imported into ArcGIS and superimposed onto the boundary of the nature reserve for the GAP analysis of *T. koraiensis*.

## 3. Results

### 3.1. Importance Analysis on Environmental Factors

**Figure 2:**
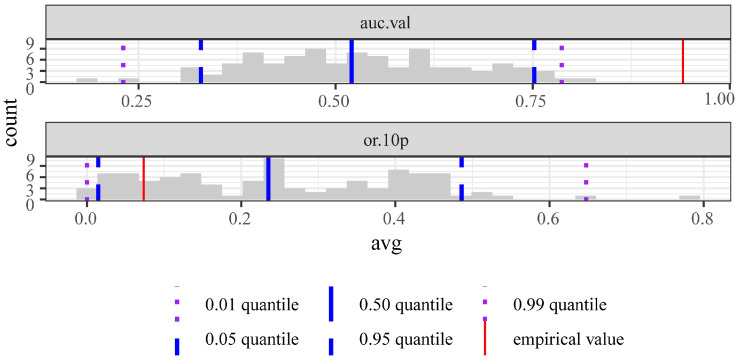
Performance comparison results of the species model and null model (auc.val: area under the curve for validation occurrences; or.10p: 10% training omission rate).

According to the AIC information criterion, when delta. AICc = 0, FC = LQHPT, and RM = 2, these are the optimal parameter combinations in this simulation. According to the comparison of the performance of the species model and the null model (Figure 2), the AUC.val (area under curve for validation occurrences) value of the species model was significantly greater than the AUC.val value of the null model, and the OR.10p (10% training omission rate) value of the species model was significantly less than the OR.10p value of the null model, thus indicating that the performance of the species model was better than that of the null model and could be used to predict the distribution of *T. koraiensis*. In this study, the average training AUC obtained from the results of 10 runs is 0.950, and the average testing AUC is 0.908, with standard deviations of 0.013 and 0.047, respectively. This indicates that the prediction results of the Maxent model are extremely good and can be further analyzed.

**Figure 3:**
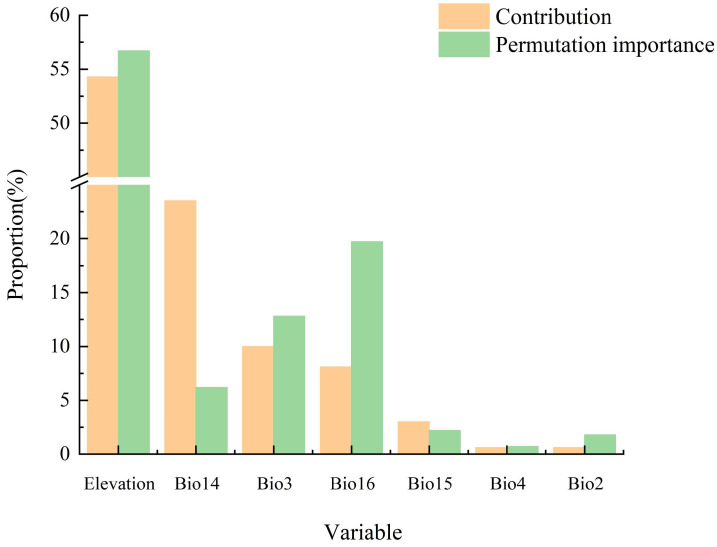
Contribution rate and permutation importance of environmental variables (Bio14: precipitation in the driest month; Bio3: isothermality; Bio16: precipitation of the wettest quarter; Bio15: precipitation seasonality; Bio4: temperature seasonality; Bio2: monthly mean temperature range).

This study combines the contribution rate and the permutation importance to determine the importance of environmental factors. Our analysis of the importance of environmental factors demonstrated (Figure 3) that, due to the differences in the assessment logic and focus points of these two methods, inconsistent rankings were obtained. The four most important environmental factors in terms of the contribution rate and permutation importance were elevation (Elevation), precipitation in the driest month (Bio14), isothermality (Bio3), and precipitation in the wettest quarter (Bio16); moreover, the cumulative contribution rate reached 95.9%. Therefore, these four environmental factors play important roles in the distribution of *T. koraiensis*.

**Figure 4:**
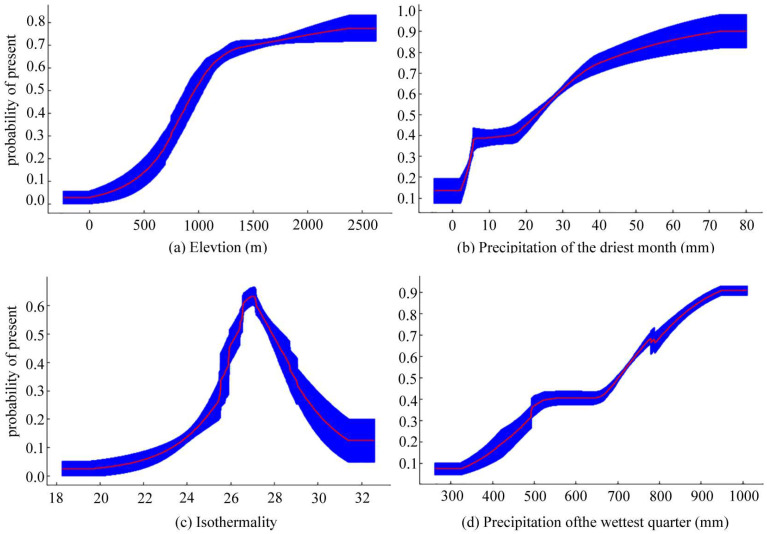
Response curves for the dominant environmental variables. (**a**) Elevation; (**b**) Precipitation of the driest month (mm); (**c**) Isothermality; (**d**) Precipitation of the wettest quarter.

The response curves of the four dominant environmental factors to the probability of *T. koraiensis* occurrence are shown in Figure 4. When the probability of occurrence is greater than 0.5, the corresponding environmental factor value is conducive to *T. koraiensis* growth [51]. As shown in Figure 4, the elevation range that is appropriate for *T. koraiensis* growth was 965–2627 m, the precipitation range in the driest month was 23–80 mm, the isothermal range was 26–30, and the precipitation range in the wettest quarter was 702–1010.

### 3.2. Prediction of Potentially Suitable Areas for T. koraiensis under Current Climatic Conditions

**Figure 5:**
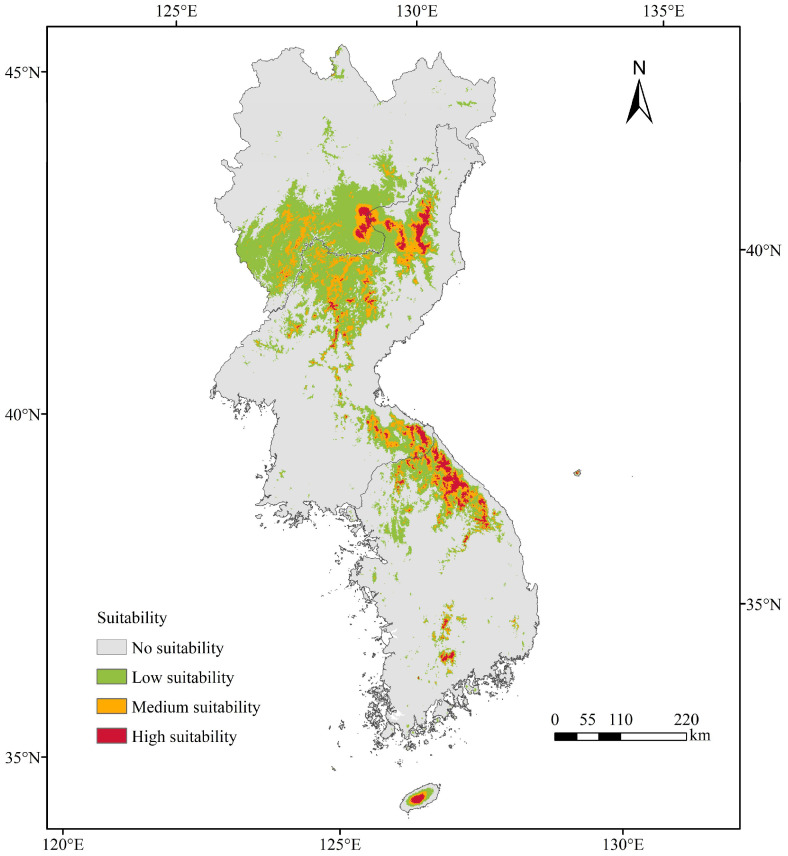
Potentially suitable area for *T. koraiensis* under current climatic conditions.

As shown in Figure 5, *T. koraiensis* was mainly distributed in southern Jilin Province, northern South Korea, and in the northern and southeastern parts of North Korea. The total suitable area for *T. koraiensis* was 7.91 × 10^4^ km^2^, which was 24.21% of the study area. The high suitability area was mainly distributed in Kumgangsan County, eastern Kangwon-do, the junction of Orang County and Paegam County, southeastern Baishan City and southeastern Yonsa County, with areas of 0.55 × 10^4^ km^2^, which was 1.68% of the total area of the study area. The medium suitability area is mainly distributed around the high suitability area, with an area of 1.75 × 10^4^ km^2^ (5.36% of total area of the study area). The total low-suitability area was 5.61 × 10^4^ km^2^ (17.17% of the total area of the study area).

### 3.3. Potentially Suitable Areas of T. koraiensis and Change in Spatial Patterns under Future Climate Scenarios

**Figure 6:**
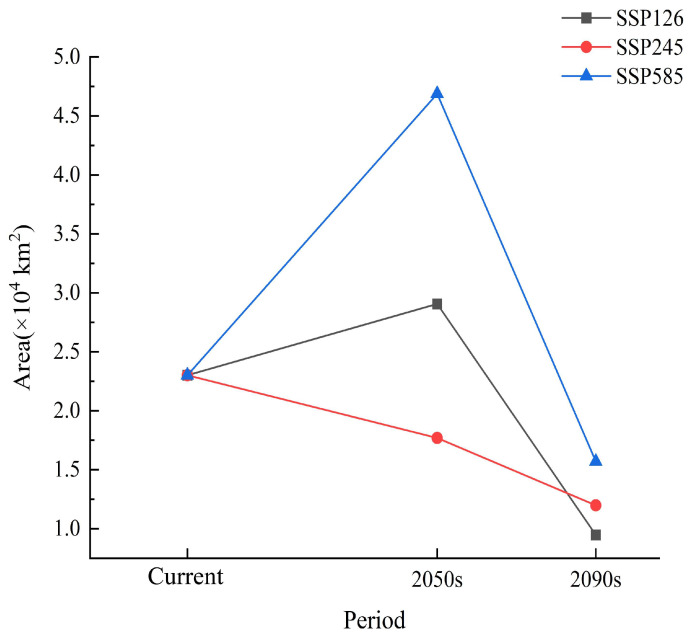
Potentially suitable areas for *T. koraiensis* under future climate scenarios.

**Figure 7:**
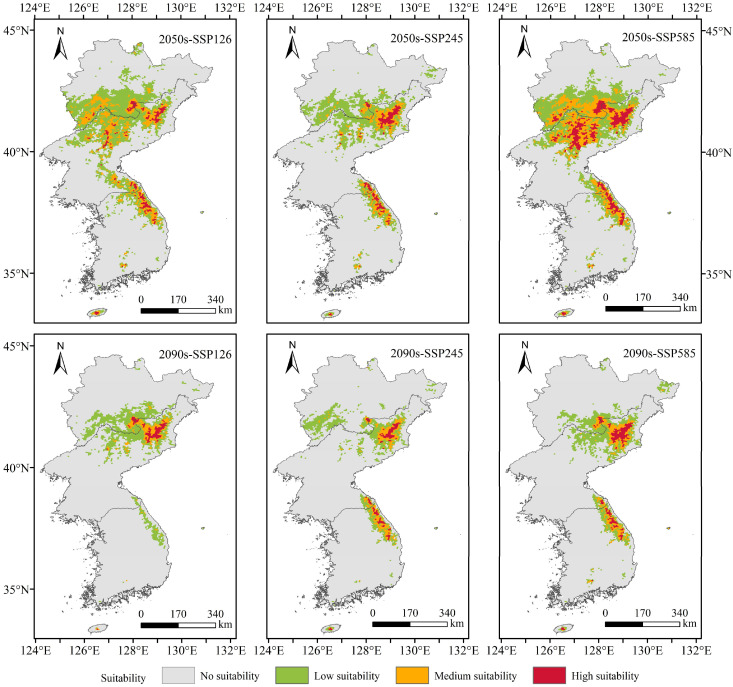
Distribution of potentially suitable areas for *T. koraiensis* under future climate scenarios.

**Figure 8:**
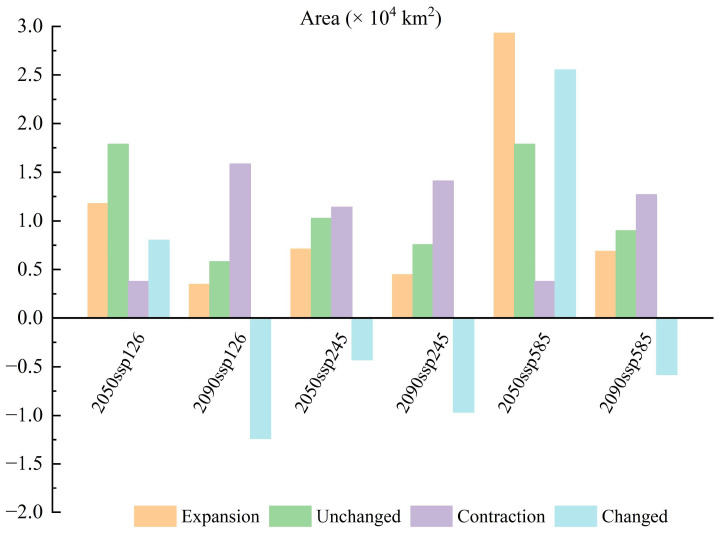
Changes in the spatial patterns of suitable *T. koraiensis* areas under diverse climate scenarios.

**Figure 9:**
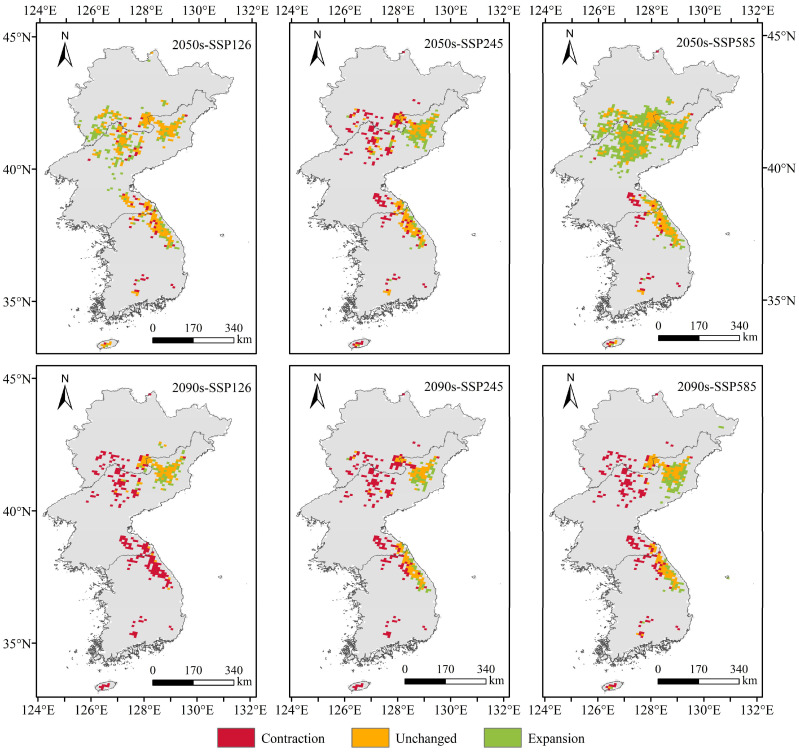
Changes in the spatial distribution of suitable areas for *T. koraiensis* under diverse climate scenarios.

Figure 6 and Figure 7 show that compared with that in the current time period, except for the continuous decrease in the total suitable area (medium-suitability area and high-suitability area) in the SSP245 climate scenario, the total suitable area of *T. koraiensis* in the SSP126 and SSP585 climate scenarios tended to initially increase and then decrease. Under the SSP126 climate scenario, the total suitable area in the 2050s slightly increased, with an increase of 1.18 × 10^4^ km^2^. In the 2090s, the total suitable area for *T. koraiensis* decreased to the highest degree, with a reduction of 1.58 × 10^4^ km^2^. The reduction area was mainly concentrated in eastern Kangwon-do, most of Kangwon-do, northeastern Chagang-do and parts of southern Jilin Province. This may be due to the decrease in precipitation in the driest quarter and the increase in precipitation in the wettest quarter; moreover, the increase exceeded the suitable range for *T. koraiensis*, thus resulting in a significant decrease in suitable habitat for *T. koraiensis*. Figure 8 and Figure 9 show that under the SSP245 climate scenario, the total suitable area in the 2050s and 2090s greatly decreased, and the reductions in area were 1.14 × 10^4^ km^2^ and 1.41 × 10^4^ km^2^, respectively. Under the SSP585 climate scenario, the total suitable area for *T. koraiensis* in the 2050s significantly increased, with an increase of 2.55. The expansion areas were mainly concentrated in Chagang-do, Yanggang-do and southern Jilin Province, and Kangwon-do was also sporadically distributed. This may have been an isothermal decline, and the precipitation in the wettest quarter was similar to the most suitable value for the growth of *T. koraiensis*, thus resulting in the expansion of the total suitable area. Although the total suitable area for *T. koraiensis* increased under the 2050s-SSP126 and 2050s-SSP585 climate scenarios, global warming will lead to a decrease in the suitable area for *T. koraiensis*, which will have a negative impact on its potential distribution.

### 3.4. GAP Analysis

**Figure 10:**
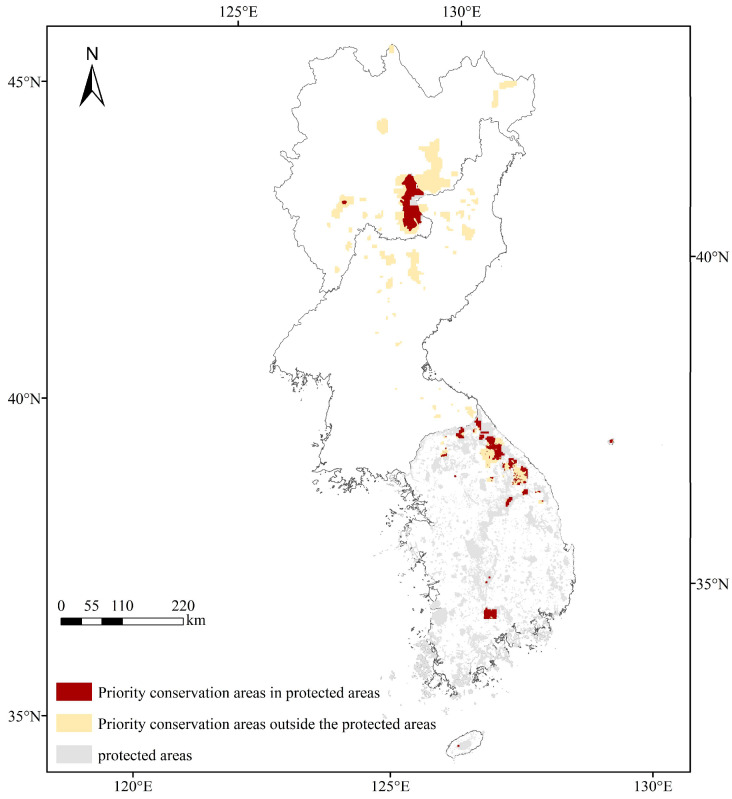
Conservation gaps for *T. koraiensis*.

The priority conservation areas that were identified by Marxan were superimposed onto the boundaries of nature reserves to construct a map of the distribution of conservation gaps (Figure 10). The discoveries demonstrated that existing nature reserves encompassed only 30.31% of the priority conservation areas of *T. koraiensis*, and 69.69% of the priority conservation areas were still unprotected, thus indicating that the existing nature reserves were not sufficient to protect *T. koraiensis*.

## 4. Discussion

### 4.1. The Dominant Environmental Factors Influencing Potential Distribution of T. koraiensis

The prediction results of the Maxent model demonstrated that elevation, precipitation of the driest month, isothermality, and precipitation of the wettest quarter were the dominant environmental factors influencing the potential distribution of *T. koraiensis*, with a cumulative contribution rate of 95.9%. According to the findings of Lan et al. [36], the analysis results of the other dominant environmental factors were essentially the same (except for precipitation seasonality and temperature seasonality). Elevation is the most important environmental factor affecting the distribution of *T. koraiensis*, which is consistent with the results of Lee et al. [52]. The Maxent model predicted that the most appropriate elevational range for the growth of *T. koraiensis* was 965–2627 m, which was consistent with the conclusion of Zhang that *T. koraiensis* is scattered at an elevation of more than 1000 m and also confirmed the biological characteristics of *T. koraiensis*. [53]. Precipitation in the warmest quarter was positively correlated with the probability of the occurrence of *T. koraiensis*. The precipitation range of 702–1010 mm is the most suitable for the growth of *T. koraiensis*, whereas drought leads to its death [53]. The optimum value of precipitation in the warmest quarter obtained by Lan et al. was 750.18 mm [36], which is not completely consistent with the results of this study. This may be due to the inconsistency of the scale of the study area. Isothermality reflects the time and amplitude of temperature change [54]. The optimum temperature value for *T. koraiensis* was 27, which indicates that this species is suitable for the distribution of *T. koraiensis* in areas with small temperature fluctuations. In summary, high elevation, stable precipitation and temperature are the key factors affecting the survival of *T. koraiensis*.

### 4.2. Influence of Climate Changes on the Geographical Distribution of T. koraiensis

In this study, the Maxent model was utilized to predict potentially suitable areas for *T. koraiensis* under recent and future climate scenarios. The results showed that the suitable area for *T. koraiensis* decreased under the three climate scenarios. This scenario may be due to the increase in greenhouse gas concentration and the obvious increase in temperature, which leads to the occurrence of extreme weather events such as drought and frequent extreme precipitation. In addition, the difficulty of seed reproduction in *T. koraiensis* and poor natural regeneration have led to a decrease in the suitable area for this species [28,55,56]. Lee et al. predicted the geographical distribution pattern of *T. koraiensis* under future scenarios by using a multimodel ensemble approach, which is also consistent with the results of this study [52]. It can be observed that it will be difficult for *T. koraiensis* to find suitable habitats in the future, and this species faces a greater threat of extinction [37]. In addition, suitable areas for *T. koraiensis* will lead to a tendency of migration to high-elevation areas. In particular, under the 2090s-SSP126 climate scenario, the elevation in the South Korean region was relatively low, and the suitable habitat area for *T. koraiensis* almost disappeared entirely. This may be due to the fact that the climate is warming and humidifying, and the original habitat conditions of *T. koraiensis* are changing. It is necessary to identify suitable habitat conditions in higher elevation areas to alleviate the adverse effects of extreme weather events. This finding is similar to the results reported for *T. koraiensis* [52], *Sambucus javanica* [57], *Agastache rugosa* [58] and *Sapindus mukorossi* [59].

The distribution data that were obtained in this study were derived from online databases, and no field surveys were conducted, which may have led to uneven or incomplete species distribution data. In addition, we did not consider the effects of photosynthetically active radiation, population competition, or the transmission capacity of the species on the geographical distribution pattern of *T. koraiensis*. In future research, these factors can be considered in modeling to improve the accuracy of the model.

### 4.3. Suggestions for the Protection of T. koraiensis

The protection and management of wild resources and habitats of *T. koraiensis* should be strengthened in the reserve area. The reserved areas are mainly distributed in Paegam County, Poch’ŏn County, Unhŭng County, southern YonsaYŏnsa County, western Samjiyon County, eastern Kangwŏn-do and southeastern Fusong County. Under threat of increasing climate warming and humidification, the reserve area may become a stable refuge for *T. koraiensis* [60]. Therefore, it is necessary to adjust the boundary of the nature reserve in this area, strengthen the protection and management of its wild resources and habitats, improve the surrounding biodiversity, and create a good breeding environment for *T. koraiensis* [53].

The edge of the increased area reduces the interference of human activities. The newly increased areas are mainly concentrated in the northern part of Hŏch’ŏn County, the eastern part of Kapsan County, a small part of the eastern part of Kangwon-do and the western part of Orang County. The increased area provides new habitats for *T. koraiensis*, which is conducive to expanding its distribution range and increasing its population size, thereby reducing the risk of extinction. Therefore, interference from human activity upon the edge of the increased area should be reduced to decrease the diffusion pressure on the *T. koraiensis* population [61].

Artificial domestication or ex situ protection should be performed in the lost area. The suitable areas for *T. koraiensis* in Hwap’yŏng County, Rangrim County, Pŏptong County, Sep’o County, Kimhyongjik County and northwestern of Kangwon-do have been reduced to varying degrees, thus indicating that the environmental conditions in these areas are no longer suitable for the survival of *T. koraiensis*. Therefore, artificial domestication research on *T. koraiensis* in this area is recommended to reduce its dependence on the natural environment and protect the wild population. In addition, in-depth research on the rapid propagation technology of *T. koraiensis* should be conducted to identify the best breeding method and to provide the necessary technical support for immigration protection [28].

Nature reserves are vital for protecting natural resources and biodiversity [62]. However, the results of the GAP analysis demonstrated that only 30.31% of the conservation priority areas were located in nature reserves, and large conservation priority areas were not effectively protected. Currently, these conservation gap areas are mainly located in the southern part of the Changbai Mountains and Kangwon-do. There are also sporadic distributions in Kangwon-do, Yanggang-do, the western part of Hamgyong-Bukto Province, and the northeastern part of Chagang-do. It is suggested that these areas be included in future systematic conservation planning.

## 5. Conclusions

In this study, the Maxent model was utilized to predict potentially suitable areas and spatial variation patterns of *T. koraiensis*, which provided a theoretical foundation for the conservation and field return of the *T. koraiensis* population. In addition, consideration of the impact of socioeconomic costs on conservation planning in the process of coupling the Maxent model and the Marxan model will effectively alleviate the contradiction between conservation and development.

The distribution of *T. koraiensis* was mainly dominated by elevation and water conditions. In the future climate scenario, the suitable area for *T. koraiensis* will decrease overall, and the high-elevation area will become a refuge for *T. koraiensis* in the future. However, if the global climate continues to warm, the suitable area in South Korea will shrink or even disappear. GAP analysis demonstrated that there is currently still a relatively large conservation gap area for *T. koraiensis*. The local government should formulate effective protection measures to strengthen the protection intensity of *T. koraiensis*. In addition, research on the genetic diversity, physiological characteristics and ecological characteristics of *T. koraiensis* should also be strengthened to explore the mechanism of *T. koraiensis* endangerment, in order to alleviate the negative impacts of global climate change.

## Figures and Tables

**Table 1:** Data source.

Data Name	Resolution	Data Source Website
Distribution data	/	Chinese Virtual Herbarium database (https://www.cvh.ac.cn/), Global Biodiversity Information Facility (https://www.gbif.org/), Documentation
Historical climate data	1 km × 1 km	Worldclim (https://worldclim.org/)
Future climate data	5 km × 5 km	Worldclim (https://worldclim.org/)
Elevation	1 km × 1 km	Worldclim (https://worldclim.org/)
Protected areas	/	Protected Planet (https://www.protectedplanet.net/)
Population density	1 km × 1 km	Worldpop (https://hub.worldpop.org/)
Road density	/	OpenStreetMap (https://www.openstreetmap.org/)
Global Impervious Surface	1 km × 1 km	Zenodo (https://zenodo.org/record/5220816, accessed on 15 December 2022)
Land utilization	30 m × 30 m	National Catalogue Service For Geographic Information (https://www.webmap.cn/mapDataAction.do?method=globalLandCover, accessed on 15 December 2022)

## Data Availability

The raw data supporting the conclusions of this article will be made available by the authors on request.
